# Endophytic *Klebsiella aerogenes* HGG15 stimulates mulberry growth in hydro-fluctuation belt and the potential mechanisms as revealed by microbiome and metabolomics

**DOI:** 10.3389/fmicb.2022.978550

**Published:** 2022-08-12

**Authors:** Ting Ou, Haiying Gao, Kun Jiang, Jing Yu, Ruolin Zhao, Xiaojiao Liu, Zeyang Zhou, Zhonghuai Xiang, Jie Xie

**Affiliations:** ^1^State Key Laboratory of Silkworm Genome Biology, Key Laboratory of Sericultural Biology and Genetic Breeding in Ministry of Agriculture, College of Sericulture, Textile and Biomass Science, Southwest University, Chongqing, China; ^2^College of Life Science, Chongqing Normal University, Chongqing, China

**Keywords:** endophyte, plant growth promotion, flood, microbiome, metabolite

## Abstract

Growth promotion and stress tolerance induced by endophytes have been observed in various plants, but their effects on mulberry regularly suffering flood in the hydro-fluctuation belt are less understood. In the present study, endophytic *Klebsiella aerogenes* HGG15 was screened out from 28 plant growth promotion (PGP) bacteria as having superior PGP traits *in vitro* and *in planta* as well as biosafety for silkworms. *K. aerogenes* HGG15 could actively colonize into roots of mulberry and subsequently transferred to stems and leaves. The 16S ribosomal RNA (V3–V4 variable regions) amplicon sequencing revealed that exogenous application of *K. aerogenes* HGG15 altered the bacterial community structures of mulberry roots and stems. Moreover, the genus of *Klebsiella* was particularly enriched in inoculated mulberry roots and was positively correlated with mulberry development and soil potassium content. Untargeted metabolic profiles uncovered 201 differentially abundant metabolites (DEMs) between inoculated and control mulberry, with lipids and organo-heterocyclic compounds being particularly abundant DEMs. In addition, a high abundance of abiotic stress response factors and promotion growth stimulators such as glycerolipid, sphingolipid, indole, pyridine, and coumarin were observed in inoculated mulberry. Collectively, the knowledge gained from this study sheds light on potential strategies to enhance mulberry growth in hydro-fluctuation belt, and microbiome and metabolite analyses provide new insights into the growth promotion mechanisms used by plant-associated bacteria.

## Introduction

The Three Gorges Reservoir (TGR), the largest reservoir ever built in China, was constructed for the purposes of flood control, hydropower generation, and navigation, where the water level is periodically maintained 145 m above sea level in summer (May–September) but increasing to 175 m in winter (October–April) (Wang et al., 2018; Yin et al., 2020). As a result of thus artificial water control, a 30 m water level fluctuation occurs on the banks, called a hydro-fluctuation belt. This annual reverse-seasonal flood maintaining nearly half a year in the TGR has caused serious degradation of the vegetation in the hydro-fluctuation belt, which has led to considerable soil erosion, habitat loss, biodiversity decline, and environmental pollution (Liu and Willison, 2013). Mulberry (*Morus* L.), a woody perennial, is a plant species with potential for revegetating the hydro-fluctuation belt of the TGR due to its strong resistance to flood stress and ability to grow well in this limited-nutrient environment (Huang et al., 2013). However, while initially vigorous, mulberry trees (“Guisangyou 62,” *Morus alba* L.) planted in 2012 at the hydro-fluctuation belt for revegetation were partially dead and their growth status varied considerably, after several years of fluctuating water levels. The well-growing newly planted trees were deeply rooted and had strong trunks and healthy leaves, while poorly-growing newly planted trees had underdeveloped root systems. Moreover, many wild mulberry trees grew exuberantly and had great vitality in the same field, which were planted by local residents and presented prior to the establishment of the TGR, but their genetic background was unclear (Xie et al., 2021). Given that the development of mulberry trees in TGR was often adversely affected by flood stress, there is a need to promote its growth and increase flood tolerance so that it can be used in revegetation efforts.

Exposure of plants to adverse environmental conditions disrupts their metabolism, ultimately leading to reduced fitness and productivity (Montesinos-Navarro et al., 2020). In the course of evolution, plants have evolved an array of protective mechanisms allowing them to adapt and survive in such stressful environments. A crucial step in plant responses is their timely perception of the stress to respond in a rapid and efficient manner. Responses typically involve activation of specific ion channels and kinase cascades and the accumulation of reactive oxygen species and phytohormones, all of which result in appropriate defense reactions and thus increase plant tolerance. Most commonly plants adjust their metabolic pathways to produce a series of anti-stress substances when encountering stress challenges. For instance, the concentrations of secondary metabolites (glycosides, alkaloids, phenolics, terpenoids) are highly accumulated in *Catharanthus roseus* (Saravanan, 2021) and *Elaeis guineensis* (Ibrahim and Jaafar, 2012) when treated with high levels of carbon dioxide. Moreover, maize specifically produced iron-benzoxazinoid complexes to defend against herbivores and further improve their growth (Hu et al., 2018).

Apart from their intrinsic mechanisms, plants also can alleviate the burden of environmental stresses by associating with particular endophytic microbes. Plant endophytes are typically non-pathogenic microbes that colonize in the interior space of plant tissues at some period in their life cycle, including roots, stems, leaves, flowers, fruits, and seeds (Compant et al., 2010; Zhang et al., 2019). They have been shown to play a crucial role in maintaining terrestrial ecosystems since their beneficial functions including defending hosts from biotic stress, alleviating harm from abiotic stress, and supporting host plant nutrition by increasing phosphorus, nitrogen, and iron levels (Bacon and White, 2015). Increased biomass in inoculated plant has been reported as a result of their colonization by a variety of endophytic genera such as *Bacillus* (Xu et al., 2019), *Streptomyces* (Jaemsaeng et al., 2018), *Klebsiella* (Zhang et al., 2017), *Pantoea* (Xie et al., 2017), and *Pseudomonas* (Han et al., 2015). In addition, the improvement of stress resistance in crop plants using endophytic microorganism is cost-effective and ecofriendly for the environment (Olanrewaju et al., 2017; Nascimento et al., 2018). For example, inoculation of endophytic *Bacillus cereus* PE31 could promote the phytoremediation efficiency of *Phytolacca acinosa* in Cadmium contaminated soils (Liu et al., 2022). Moreover, introduction of exogenous endophytes can induce host plants to recruit beneficial taxa. The process alters associated-microbiome composition of host, thus indirectly attributes to plant development as well (Welmillage et al., 2021). The application of endophytic *Rhizobium* sp. RF67 isolated from *Vaccinium angustifolium* resulted in root-associated bacteria variation, which boosted cooperation of plant-growth-promoting endophytes (Yurgel et al., 2022). Moreover, *Piriformospora indica* recruited the member of Firmicutes and decreased Proteobacteria in rice roots, resulting in enhancement of rhizosheath and improvement of drought tolerance (Xu F. Y. et al., 2022). Beneficial interactions between endophyte and plant have gradually become a focus of many studies addressing ecological restoration such as deserts (Jain et al., 2021), saline-alkaline lands (Lu et al., 2021), and oil or heavy metal contaminated lands (Liu et al., 2022). However, the utilization of endophyte and plant to restore ecosystems subjected to repeated flood stress such as the hydro-fluctuation belt of the TGR has not received much attention.

The results of 16S ribosomal amplicon sequencing in our previous study revealed that the complexity of endophytic bacterial interactions in well-growing mulberry trees, including newly planted mulberry and wild mulberry, was higher than it was in poorly-growing mulberry tree. Additionally, well-growing trees were found to have recruited similar endophytic bacteria that enabled them to flourish in hydro-fluctuation belt (Xie et al., 2021). Thus, we hypothesized that endophytic bacteria of well-growing mulberry trees could be a potential resource to promote mulberry growth in such settings. Therefore, objectives of this work were to (i) obtain endosphere-derived bacteria from well-growing mulberry trees and detect their potentials to benefiting the growth of mulberry *in vitro* and *in planta*; (ii) test plant colonization capability of plant growth promoting bacteria by using of a green fluorescence protein marker; and (iii) further decipher the underlying preliminary mechanisms how bacteria promote mulberry growth by comprehensively interrogating the mulberry associated microbiome and metabolome.

## Materials and methods

### Sample collection and isolation of endophytic bacteria

Samples were collected from well-growing mulberry trees including wild mulberry trees (WM) and newly planted mulberry trees (NM) from the hydro-fluctuation belt in Longjiao Town, Yunyang County, Chongqing Municipality, China (30°49′26′′ N, 108°52′14′′ E) in May, 2020. A total of four types of sample were obtained including two compartments (stem and root) of WM and NM. Three biological replicates were performed for each sample. Characteristics of mulberry trees were shown in Supplementary Table 1. These mulberry trees were suffered flood stress lasting for about 6 months. The elevation of this sampling area was approximately at 172 m above sea level and the water level of TGR was about 152 m above sea level at that time. Mulberry stem segments were collected at approximately 25 cm in length where leaves and small side branches had been removed. The sampling depth of root was about 15 cm below soil surface and the length of root segments was about 20 cm. All samples were immediately transported back to the laboratory and stored at 4°C until further processing.

Surface sterilization of the mulberry stem and root was performed according to a previously described procedure (Xu et al., 2019), and the endophytic bacteria were isolated using the fragmentation technique (Liotti et al., 2018). Samples were washed with tap water to remove soil and other debris before being cut into pieces with 3.0–5.0 cm in length. The samples were then thoroughly soaked in 75% ethanol and rapidly flame-sterilized. Afterward, samples were peeled to obtain smaller fragments and placed on nutrient agar (NA), Gause’s agar (GA), Luria-Bertani agar (LB), and Trypticase Soya agar (TSA) medium at 28°C for 5 days. Isolates with different morphological characteristics were purified and stored with 50% glycerol at −80°C.

### Screening of plant growth promoting bacteria and molecular characterization analysis

The plant growth promotion (PGP) traits of endophytes, including phosphate (P) solubilization, indole-3-acetic acid (IAA), siderophore, and acetoin production, and antagonistic activity against phytopathogen *Sclerotinia sclerotiorum*, which could seriously infect mulberry flowers and branches, were qualitatively determined by following procedures. Specifically, isolates were inoculated in LB medium and incubated at 30°C with 180 rpm for 16 h, and then 10 μL of each bacterial culture was inoculated on different medium to detect PGP traits, respectively. The ability of isolates to P solubilization was evaluated in Pikovskaya’s agar medium containing tricalcium phosphate (Gaind, 2016) and the siderophore activity was tested in chrome azurol-s agar medium (Jasim et al., 2013). The detections of IAA and acetoin activities were conducted in LB medium as described by Patten and Glick (1996) and Vivijs et al. (2014), respectively. The antifungal bioactivities were evaluated on PDA medium by the dual culture technique as described by Xu et al. (2019).

Molecular characterization of plant growth promoting bacteria (PGPB) was tested based on 16S rRNA gene sequence. The genomic DNA of PGPB was extracted using a PrepMan Ultra Sample Preparation Reagent kit (Applied Biosystems, Palo Alto, CA, United States) according to the manufacturer’s instructions and amplified by PCR using universal 16S rRNA gene primers 27F/1492R (Ou et al., 2022). Each 25 μL PCR mixture contained 12.5 μL of 2 × Rapid Taq Mater Mix (Vazyme, Nanjing, China), 1 μL of each primer (10 μM), and 10 ng of template DNA. The PCR reaction was carried out using the following conditions: 1 cycle of 95°C for 4 min; followed by 30 cycles of 94°C for 30 s, 55°C for 45 s and 72°C for 1 min; and a final extension at 72°C for 8 min. The PCR amplified products were purified with the DNA Clean & Concentrator™-5 Kit (Zymo Research, United States) and then sequenced by the Sanger method at Sangon Biotechnology Co., Ltd., Shanghai, China. The nucleotide sequences of PGPB were compared using Basic Local Alignment Search Tool (BLAST) method and deposited in NCBI GenBank database. In present study, the isolates with high level of identity (97–100%) were selected as the closest match, and all isolates were classified to the genus level.

### Evaluation of the activity of the bacteria in mulberry

The isolates exhibiting high PGP potentials *in vitro* were selected and their effects on mulberry seedling growth were further evaluated *in planta*. Fresh colony of PGPB was inoculated into LB medium and incubated at 30°C with 180 rpm for 18 h. The cultures were centrifuged at 8000 rpm for 10 min and precipitate was adjusted at 1 × 10^7^ CFU/mL using the sterile water (Ou et al., 2022).

“Guisangyou 62,” a variety of white mulberry, is a potential for phytoremediation since the quick-growth and ecological-adaptation characterizations (Zhao et al., 2013; Jiang et al., 2019). This mulberry cultivar thus was applied in the present study. Mulberry seeds were surface sterilized with 75% ethanol for 3 min and 10% sodium hypochlorite for 3 min. After five times rinse with sterile distilled water, seeds were placed in a 9-cm-diameter petri dish containing sterilized moistened filter paper and maintained in growth chamber at 25°C with a photoperiod of 12 h at 200 μmol⋅m^–2^⋅s^–1^ and 70% humidity. At two-leaf stage, seedlings were transferred to pot which was filled with mixtures of humus and filed soil (4:1; *v*/*v*) collected from Southwest University experimental farm (29°49′1″ N, 106°24′57″ E). The mixed soils were sterilized four times by autoclaving at 121°C for 2 h (Li et al., 2012; Cao et al., 2013). Each pot contained three mulberry seedlings with similar size and height. After 2 weeks, mulberry seedlings with three-leaf were inoculated with PGPBs through irrigating 30 mL of bacterial suspension into soils in each pot, and the mulberry seedlings treated with sterile water were served as control. Each treatment was replicated twenty times. After 30-day inoculation, eight seedlings were randomly selected in each treatment to measure length of root and shoot, fresh and dry weight of root and shoot, and number of root tip.

To assess the effects of PGPBs on mulberry growth under flood stress condition, the remaining mulberry seedlings were completely submerged wherein the water level was 30 cm above soil surface. After 40 days, the degree of cell death of mulberry leaf was determined using Evan’s blue staining (Taylor and West, 1980). Finally, nine seedlings were randomly selected and the root fresh weight was calculated, and the total length and fork of roots were analyzed using the GXY-B root analysis system (Hangzhou Lvbo instrument Co., Ltd, Hangzhou, China).

### Bio-safety assessment of plant growth promoting bacteria on silkworms

In order to lay a good foundation for utilization of PGPB to promote mulberry growth in the field, the biosafety was studied through feeding silkworms with PGPBs-infected mulberry leaves. One milliliter of 1 × 10^7^ CFU/mL bacterial suspension was sprayed on the surface of each leaf (Xie et al., 2017). The sterile water was used as control. Mulberry leaves dried naturally were applied to feed healthy silkworm larvae (871 × 872 strain) at the 4th instar with two times each day. Afterward, all silkworms were fed with clean mulberry leaves until cocoon formation. Each treatment consisted of three replicates and each replicate contained forty silkworms. The growth status of silkworm was observed every day. The survival rate and rate of cocoon formation of three replicates were determined. Forty silkworms were randomly selected to measure cocoon weight, cocoon shell rate and weight, and pupal weight.

### Identification of HGG15 strain

Based on plant growth promoting activity in mulberry seedlings and bio-safety on silkworm of PGPBs, the HGG15 strain was finally screened out for further study. The morphological characteristics of HGG15 in LB incubated at 37°C for 24 h were recorded and further observed in scanning electron microscope (Hitachi, SU3500, Japan). Gram staining was performed and observed under an optical microscope (Becerra et al., 2016). Series of biochemical tests such as Voges-Proskauer and motility test were conducted using an HK-MID-66 kit (HUANKAI, China) following the protocol provided by the manufacturer. Phylogenetic tree of HGG15 based on 16S rRNA gene sequence was constructed by applying the neighbor-joining method using MEGA version 6.0 with 1,000 replicates of bootstrap values (Tamura et al., 2011).

### Colonization of mulberry by HGG15 strain

To explore colonization characteristics of HGG15 strain in mulberry, the plasmid pGFP4412 containing green fluorescence protein (*gfp*) and kanamycin resistance gene was transformed into the wild type HGG15 strain by electroporation technique. The competent cells were prepared in 10% glycerol and electroporated at 2.5 kv for 5 ms. Transformed colonies that emitted green fluorescence stably under fluorescence microscopy (Leica, DM3000, Germany) were obtained and designated HGG15/gfp strain.

The cultivation of mulberry seedlings was shown as described above and roots of mulberry at two-leaf-stage were immersed in 1 × 10^7^ CFU/mL bacterial suspension for 4 h and then re-planted into the sterile soil. The sterile distilled water was served as control group. Afterward, seedlings were removed from pots, surface disinfected using 75% ethanol for 1 min, and separated into roots, stems and leaves at different time after inoculation. And then, these tissues of mulberry were cut into small pieces to observe the colonization using fluorescence microscopy. In addition, these tissues were weighed and ground in 1 mL sterile distilled water, and serially diluted and plated on LB plates supplemented with 50 μg/mL kanamycin to count the number of colony. Three replicates were used for each treatment.

### Detection of mulberry associated bacterial communities after HGG15 inoculation

To explore the potential mechanism of HGG15 strain to promote mulberry growth whether attributed to variation of host-associated microbiome, the rhizosphere soil, root, and stem of mulberry were collected after 30-day inoculation. A total of nine individual seedlings were randomly selected and three seedlings mixed as one replicate. The rhizosphere soils adhering to the mulberry root approximately 1 mm were obtained using a sterile scalpel. And then, approximately 5 cm roots and stems were collected for each mulberry seedling, and washed with tap water to remove soil and other debris. The root and stem samples were thoroughly soaked in 75% ethanol and rapidly flame-sterilized and stored at −80°C for further processed.

The DNA of all samples was extracted using FastDNA^®^ Spin Kit (MP Bio, Santa Ana, CA, United States) (Xu F. Y. et al., 2022) according to manufacturer’s instructions with the moderated modification. Briefly, approximately 0.5 g of stems and roots were homogenized in liquid nitrogen. The prepared stem and root tissues and rhizosphere soil were transferred into Lysing Matrix E Tubes. And then, sodium phosphate buffer and MT buffer were added and supernatants were collected by centrifugation (12,000 rpm, 10 min). Then, 250 μL of PPS was added in tube and centrifuged at 12,000 rpm with 5 min. The upper phase was then collected and transferred to a new tube and 1 mL binging matrix suspension was added. Afterward, 600 μL mixtures were transferred into SPIN™ Filter tubes and subsequently centrifuged. Finally, 500 μL SEMW-M was added into SPIN™ Filter tubes and centrifuged (12,000 rpm, 2 min), and DNA was eluted using 100 μL DES. The DNA was checked on 1% agarose gel, and DNA concentration and purity were determined with NanoDrop 2000 UV-vis spectrophotometer (Thermo Scientific, Wilmington, NC, United States). The bacterial 16S rRNA gene was amplified with primer pairs 338F (5′-ACTCCTACGGGAGGCAGCA-3′) and 806R (5′-GGACTACHVGGGTWTCTAAT-3′) (Ou et al., 2019) by an ABI GeneAmp 9700 PCR thermocycler (ABI, CA, United States). Each 20 μL PCR mixture contained 4 μL of 5 × FastPfu Buffer, 2 μL of 2.5 mM dNTPs, 0.8 μL of each Primer (5 μM), 0.4 μL of FastPfu Polymerase and 10 ng of template DNA. The PCR reactions were conducted using the following program: 3 min of denaturation at 95°C, 27 cycles of 30 s at 95°C, 30 s for annealing at 55°C, and 45 s for elongation at 72°C, and a final extension at 72°C for 10 min. Sterile water was served as negative control sample to avoid potential microbial contaminants in the DNA extraction and amplification processes. The PCR product was extracted from 2% agarose gel and purified using the AxyPrep DNA Gel Extraction Kit (Axygen Biosciences, Union City, CA, United States) according to manufacturer’s instructions. Purified amplicons were pooled in equimolar and paired-end sequenced (2 × 300) on an Illumina MiSeq platform (Illumina, San Diego, CA, United States) by Majorbio Bio-Pharm Technology Co., Ltd. (Shanghai, China). Pairs of reads were spliced into a sequence according to the direct overlap relationship of paired-end reads. At the same time, the quality of reads and splicing effect were controlled and filtered, and correction of the sequence direction was made according to the end of the box sequence. Finally, the high-quality sequences obtained after filtering were assigned to samples according to barcodes. The raw reads were deposited into the NCBI Sequence Read Archive (SRA) database.

Operational taxonomic units (OTUs) with 97% similarity cutoff (Edgar, 2013) were clustered using UPARSE (version 7.0.1090), and chimeric sequences were identified and removed by VSEARCH (version 1.0.10). The taxonomy of each OTU representative sequence was analyzed using the BLAST algorithm with Silva database (release138^1^) against the bacterial 16S rRNA genes (Pruesse et al., 2007; Quast et al., 2013). Each sample was rarefied to 2000 reads (Beckers et al., 2017). The sequences classified as cyanobacteria and mitochondria were removed from the OTU table. The filtered table was used for further analyses. The α-diversity including Shannon and Sobs indices was calculated using Mothur software (version 1.30.2) at a 97% identity level (Schloss et al., 2009). The different significances of α-diversity among samples were compared based one-way analysis of variance (ANOVA). Bacterial community structures were analyzed at different classification levels using R software (version R-3.3.1). The Venn diagram was generated using R script and principal coordinates analysis (PCoA) was conducted based on Bray–Curtis distances at OTU level. The comparison of endophytic bacterial abundance was analyzed using Wilcoxon rank-sum test based on genus level. Moreover, Spearman correlation coefficient of the top 20 abundant bacterial genera and environmental factors including mulberry growth parameters and soil properties was calculated and displayed on the heat map (Zhou et al., 2017).

### Assessments of soil biochemical properties

Approximately 50 g soil samples were collected from each pot containing mulberry for detection of the biochemical properties after 30-day inoculation, and each treatment consisted of three replicates. Specifically, soils were dried at 105°C and filtered by a 2 mm sieve. Afterward, the soil sample was subjected to analyze organic carbon (OC), organic matter (OM), available phosphorus (AP), total potassium (TK), available potassium (AK), and available iron (Fe). Soil properties were analyzed using standard soil test methods as described by the agriculture protocols (Lu, 2000; Zhou et al., 2017). Soil OM, TP, and TK were determined by the dichromate oxidation process, Mo-Sb anti spectrophotometric method, and atomic absorption spectrophotometry, respectively. AP was extracted with NH_4_F-HCl solution, and then determined by ultraviolet visible spectrophotometer. AK was extracted with 1 M NH_4_OAc, and then determined by flame absorption spectroscopy. Fe was extracted with DTPA-CaCl_2_-TEA, and then determined by atomic absorption spectrophotometry.

### Analysis of mulberry root metabolites

To understand if variations in host metabolism were correlated with HGG15 strain, metabolomics of mulberry root were employed after 30-day inoculation. A total of 18 individual mulberry seedlings were collected in each treatment and three seedlings were randomly mixed as one replicate. Six biological replicates were performed for each treatment. The entire roots were washed with tap water to remove soils and 50 mg of roots were accurately weighed, and then the metabolites were extracted using 400 μL of 80% methanol solution containing 2 mg/L L-2-chlorophenylalanine. The mixture was settled at −20°C and processed by a high-throughput tissue crusher Wonbio-96c (Wonbio Biotechnology, Shanghai, China) at 50 Hz for 6 min, then vortexed for 30 s and ultrasonically treated at 40 Hz for 30 min at 5°C, followed by precipitation for 30 min at −20°C and centrifugation at 13,000 rpm at 4°C for 15 min. The supernatants were then transferred into vials for subsequent ultra-performance liquid chromatography-tandem mass (UHPLC-MS/MS) spectrometry. A quality-control sample was prepared by mixing 20 μL of each sample in order to control the accuracy and stability of the method.

Samples were separated on Thermo UHPLC plat equipped with an Acquity Beh C18 column (2.1 mm × 100 mm i.d.; 1.7 μm; Waters, Milford, DE, United States) by Majorbio Bio-Pharm Technology Co., Ltd. (Shanghai, China). Mobile phase A was 0.1% formic acid in water, and phase B was 0.1% formic acid in acetonitrile: isopropanol (1: 1, *v*/*v*). The sample injection volume was 2 μL, and the flow rate was set to 0.4 mL/min with a column temperature of 40°C. The gradient condition is as follows: from 0 to 3 min, 5–20% (B); from 3 to 9 min, 20–95% (B); from 9 to 13 min, 95% (B); from 13 to 13.1 min, 95–5% (B), from 13.1 to 16 min, 5% (B) for equilibrating the systems. The mass spectrometric data were collected using a Thermo UHPLC-Q Exactive Mass Spectrometer equipped with an electrospray ionization source operating in either positive or negative ion mode. The optimal parameters were set as follows: scan type: 70–1050 m/z; sheath gas flow rate: 40 psi; aux gas flow rate: 30 psi; aus gas heater temperature: 400°C; capillary temperature: 320°C; ion-spray voltage floating, −2.8 kV in negative mode and 3.5 kV in positive mode, respectively; resolution: 17500 (MS^2^). Data acquisition was performed with the Data Dependent Acquisition mode.

The raw data were imported into the Progenesis QI 2.3 (Nonlinear Dynamics, Waters, United States) for peak detection and alignment. The preprocessing results generated a data matrix that consisted of the retention time, mass-to-charge ratio values, and peak intensity. The normalized data were used to predict the molecular formula based on additive ions, molecular ion peaks and fragment ions. Then, peaks were searched in human metabolome database (HMDB)^2^ and Metlin database^3^ for metabolite identification. Orthogonal partial least squares discriminate analysis (OPLS-DA) and principal components analysis (PCA) analysis were used to determine global metabolic changes. The *P*-values were estimated with paired Student’s *T*-test on single dimensional statistical analysis. Variable importance in projection (VIP) of metabolites represented their contributions to the global metabolic changes and the metabolites with VIP > 1 and *P*-value < 0.05 were considered to be differential metabolites. The functions of these metabolites and metabolic pathways were studied using scipy (Python packages)^4^ based on the Kyoto Encyclopedia of Genes and Genomes (KEGG) database. The heatmap of correlation between top 40 metabolites of mulberry root and bacterial genera was generated using the Pearson correlation coefficient.

### Statistical analysis

All data were performed using SPSS Statistics (Version 17.0. Chicago, IL, United States). Differences between treatments in the mulberry seedling and mulberry associated-bacterial diversity were analyzed using Tukey’s one-way ANOVA. The statistical significances of silkworm growth and soil properties were analyzed using *T*-test. ^***^, ^**^, and * indicated significant difference at *P* < 0.001, *P* < 0.01, and *P* < 0.05, respectively.

## Results

### Isolation of endophyte and screening of plant growth promoting bacteria

A total of 343 endophytic bacteria were isolated from mulberry plants. 119 and 88 isolates were obtained from stems and roots of wild mulberry, respectively, while 68 and 68 isolates were obtained from stems and roots of newly planted mulberry, respectively (Supplementary Table 2). Among them, 28 isolates exhibited phenotypes commonly observed bacteria capable of plant growth promotion, including the ability to solubilize P, produce IAA, siderophores, and acetoin, and exhibit antagonism against *Sclerotinia sclerotiorum* as revealed by the presence of an inhibition zone (Table 1), and their near full-length 16S rRNA gene sequences were deposited in the GenBank under accession numbers ON786677-ON786703 and ON090422. A total of 25 and 27 isolates possessed P-solubilization and IAA-producing capability, respectively. Moreover, 16 and 25 isolates had the ability to produce siderophores and acetoin, respectively, and fifteen PGPBs had anti-fungal capability *in vitro*. Ultimately, *Enterobacter* sp. HLG5, *Lelliottia* sp. HTJ13, *Pantoea* sp. HLJ21, and *Klebsiella* sp. HGG15 strains were screened as those strains with the highest potential for plant growth promotion based on these traits *in vitro*.

**TABLE 1:** Characterization of endophytic bacteria for potential plant growth-promoting traits.

Strains	P-solubilization (cm)	IAA (mg/mL)	Siderophores (cm)	Inhibition zone (cm)	Acetoin (μg/mL)
*Enterobacter* sp. HLJ6-2	0.90	7.15	—	0.23	—
*Enterobacter* sp. HGJ7-2	0.67	19.75	—	—	101.23
***Enterobacter* sp. HLG5**	0.80	2.76	0.29	0.30	33.87
*Atlantibacter* sp. HLJ20	—	6.25	0.12	—	223.44
*Atlantibacter* sp. HLJ10	0.90	2.66	0.36	—	200.35
*Pantoea* sp. HLJ16-1	0.70	6.11	—	0.31	200.35
*Kosakonia* sp. HLJ19-2	0.60	6.01	—	—	370.67
*Leclercia* sp. HNG10-2	0.27	7.37	—	—	164.74
***Lelliottia* sp. HTJ13**	0.17	3.53	0.24	0.13	363.93
*Rahnella* sp. HNJ30	0.13	18.49	0.44	—	254.23
*Pantoea* sp. HGJ8	0.80	13.92	—	—	33.87
*Pantoea* sp. HGJ11	—	11.74	0.36	—	—
*Pantoea* sp. HTJ6	0.33	5.91	0.51	0.43	32.91
***Pantoea* sp. HLJ21**	0.60	10.96	0.17	1.33	76.21
*Pantoea* sp. HTJ25	0.73	5.86	0.40	—	87.76
*Pantoea* sp. HNJ8	1.03	8.64	0.25	1.20	165.70
*Pantoea* sp. HNJ21	0.20	6.01	0.50	0.36	65.63
*Pantoea* sp. HNJ32	0.37	13.05	—	—	32.91
*Pantoea* sp. HNG24	0.93	4.8	—	0.63	85.84
*Klebsiella* sp. HNG29	—	3.39	0.20	0.30	411.09
*Klebsiella* sp. HLG11	0.67	4.8	—	0.87	69.48
*Klebsiella* sp. HLG12	0.97	2.51	—	—	19.44
*Klebsiella* sp. HLG15	0.67	5.38	0.10	0.10	223.44
*Klebsiella* sp. HLG29	0.97	37.56	0.23	—	157.04
***Klebsiella* sp. HGG15**	0.90	6.69	0.38	0.40	363.93
*Klebsiella* sp. HGG18	1.10	3.44	—	0.80	273.48
*Klebsiella* sp. HGG20	1.33	—	—	—	—
*Klebsiella* sp. HTG12	0.60	3.1	0.14	0.60	61.78
Total	25	27	16	15	25

The bold isolates were selected as potential PGPBs since their great growth-promoting traits.

### Effect of plant growth promoting bacteria on mulberry growth and their bio-safety for silkworms

**FIGURE 1:**
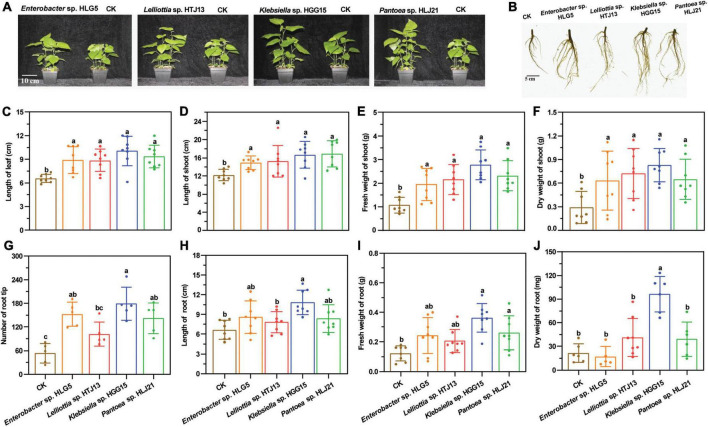
The growth promotion ability of potential four PGPBs on mulberry seedlings after 30-day inoculation. **(A)** The growth status of mulberry applied with the 1 × 10^7^ CFU/mL bacterial suspensions. **(B)** The representative photograph of mulberry root architectures. **(C)** Length of mulberry leaf. **(D)** Length of mulberry shoot. **(E)** Fresh weight of mulberry shoot. **(F)** Dry weight of mulberry shoot. **(G)** Number of mulberry root tip. **(H)** Length of mulberry root. **(I)** Fresh weight of mulberry root. **(J)** Dry weight of mulberry root. Values represented the mean ± standard deviation of replicates (*n* = 8). Different letters indicated statistical differences using Tukey’s one-way ANOVA (*P* < 0.05).

To determine the effect of these four bacteria on mulberry growth, bacterial suspensions were inoculated into mulberry seedlings. Co-cultivation experiments revealed that application of these strains had a large positive impact on mulberry shoot (Figure 1A) and root growth (Figure 1B). The four strains significantly promoted length of mulberry leaf (Figure 1C) and shoot (Figure 1D) (*P* < 0.05) compared with control plants. A corresponding significant increase of fresh weight of mulberry shoots was also observed in endophyte-treated plants (*Enterobacter* sp. HLG5: 81.51%; *Lelliottia* sp. HTJ13: 100.37%; *Klebsiella* sp. HGG15: 158.83%; *Pantoea* sp. HLJ21: 115.89%) (Figure 1E). In addition, mulberry seedlings treated with PGPBs had a higher dry weight of mulberry shoots than controls, increasing by 117.15, 147.82, 184.19, and 122.68% in *Enterobacter* sp. HLG5, *Lelliottia* sp. HTJ13, *Klebsiella* sp. HGG15, and *Pantoea* sp. HLJ21 group, respectively (Figure 1F). All of the bacterial isolates enhanced shoot growth, but differed in their stimulation of the roots of mulberry. The number of root tips of mulberry inoculated with *Enterobacter* sp. HLG5 (64.87%), *Klebsiella* sp. HGG15 (235.21%), and *Pantoea* sp. HLJ21 (165.92%) strains was greatly increased compared to control (*P* < 0.05) (Figure 1G). Moreover, *Klebsiella* sp. HGG15 significantly increased root length (63.28%) (Figure 1H), fresh weight (195.92%) (Figure 1I), and dry weight (348.79%) (Figure 1J).

**FIGURE 2:**
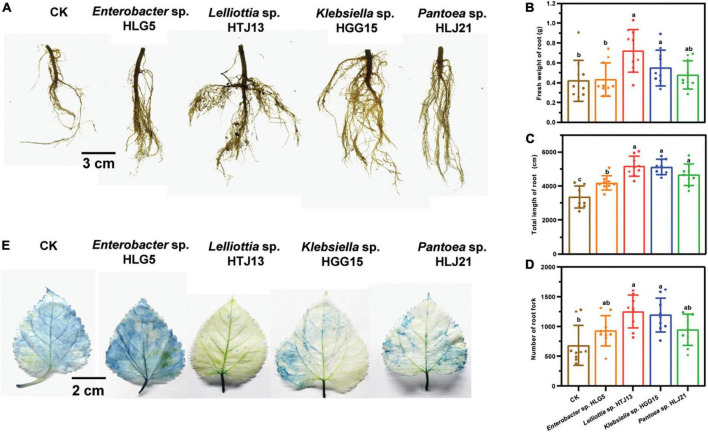
Effect of potential four PGPBs on mulberry flood tolerance. **(A)** Root architecture of mulberry after suffering flood stress. **(B)** Fresh weight of mulberry root. **(C)** Total length of mulberry root. **(D)** Number of mulberry root fork. **(E)** Representative photograph of the damage of mulberry leaf suffering flood stress by Evan’s blue staining. Values represented the mean ± standard deviation of replicates (*n* = 9). Different letters indicated statistical differences using Tukey’s one-way ANOVA (*P* < 0.05).

Additionally, the mulberry roots inoculated with these four strains exhibited greater vigor after 40 days of flood stress (Figure 2A). Specifically, root biomass (30.48 and 71.43%) (Figure 2B), total root length (52.61 and 53.74%) (Figure 2C), and the number of root fork (74.23 and 82.55%) (Figure 2D) were significantly higher in plants treated with *Klebsiella* sp. HGG15 and *Lelliottia* sp. HTJ13 under flood condition, respectively. Moreover, Evan’s blue staining to reveal dead tissues indicated leave cell death in mulberry treated with *Klebsiella* sp. HGG15, *Lelliottia* sp. HTJ13, and *Pantoea* sp. HLJ21 were slighter than in control plants or plants treated with *Enterobacter* sp. HLG5 (Figure 2E). Together, these results suggested that both *Klebsiella* sp. HGG15 and *Lelliottia* sp. HTJ13 had the greatly potential ability to relieve the negative impacts of flood stress on mulberry.

The potential use of PGPBs in the hydro-fluctuation belt of the TGR would require demonstration that they would not interfere with the down-stream uses of mulberry, such as in silk production. The impacts of PGPBs were thus evaluated by feeding them to silkworms. The growth of silkworms fed at the fifth instar and pupal stages presented did not differ from that of control insects (Supplementary Figure 1A). The PGPBs had no negative impacts on whole cocoon weight, pupal weight, cocoon shell weight, and rate of cocoon shell development in silkworms (Supplementary Figures 1B–E). Although the *Enterobacter* sp. HLG5 significantly enhanced whole cocoon weight (16.21%), pupal weight (16.23%), and cocoon shell weight (20.33%) of the survival silkworms (Supplementary Figures 1B–E), the survival rate (53.3%) (Supplementary Figure 1F) and cocoon production rate (65.0%) (Supplementary Figure 1G) of silkworms were significantly decreased in the insects fed *Enterobacter* sp. HLG5, and bodies of the dead silkworms were black and soft. Overall, these results revealed that *Enterobacter* sp. HLG5 strain was harmful to silkworms, while *Lelliottia* sp. HTJ13, *Klebsiella* sp. HGG15, and *Pantoea* sp. HLJ21 all exhibited no toxicity to silkworms.

### Characterization of HGG15 strain

**FIGURE 3:**
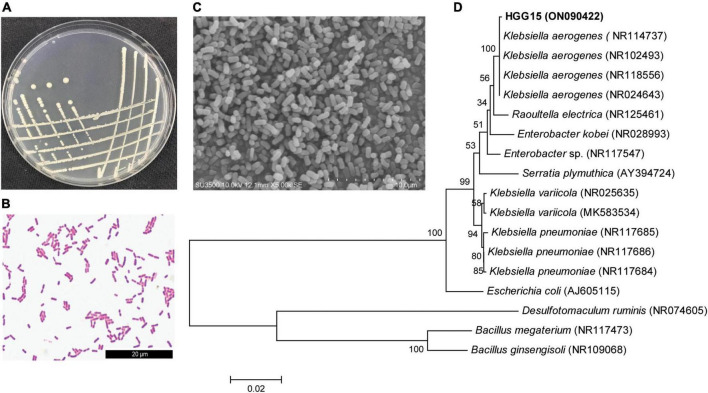
Characterization of *Klebsiella* sp. HGG15 strain. **(A)** Colony feature on LB medium after 24 h. **(B)** Gram-positive staining. **(C)** Scanning electron microscopy of bacterial cells. **(D)** Phylogenetic tree based on 16S rDNA. The tree was constructed by MEGA 6.0 using neighbor-joining analysis of 1,000 bootstrap replications.

*Klebsiella* sp. HGG15 strain was ultimately selected for further study since it exhibited the plant growth promoting traits *in vitro* and *in planta* as well as posing no bio-safety concerns for silkworms. Colonies of *Klebsiella* sp. HGG15 strain were white on LB medium (Figure 3A). Gram staining and scanning electron microscope results showed it was a short rod and gram-negative bacterium (Figures 3B,C). Meanwhile, the strain produced hydrogen sulfide and nitrate reductase, but was negative for utilization of urea and lactose (Supplementary Table 3). Moreover, phylogenetic analysis based on full length 16S rDNA indicated that it was identical to *Klebsiella aerogenes* (Figure 3D), being consistent with its morphological characteristics, biochemical, and molecule characteristics.

### Colonization and population dynamics of *Klebsiella aerogenes* HGG15/gfp in mulberry

**FIGURE 4:**
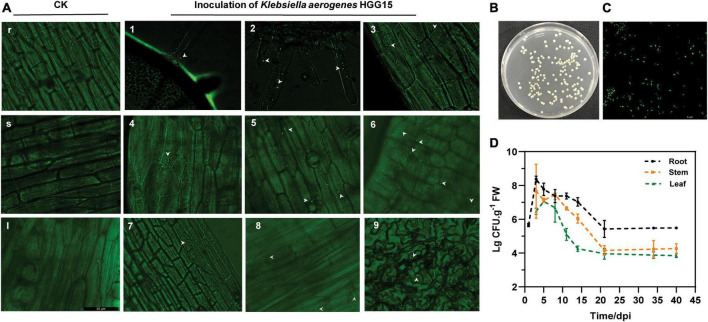
Colonization and population dynamics of gfp-tagged *K. aerogenes* HGG15 in mulberry seedlings. **(A)** Infection and colonization of gfp-tagged *K. aerogenes* HGG15 in mulberry. r, mulberry root of control group in 1 dpi; s, mulberry stem of control group in 3 dpi; I, mulberry petiole of control group in 7 dpi; 1, mulberry root hair of inoculation group in 1 dpi; 2, mulberry lateral root of inoculation group in 1 dpi; 3, mulberry root epidermis of inoculation group in 2 dpi; 4–6, mulberry cortex of inoculation group in 3–5 dpi; 7–8, mulberry petiole of inoculation group in 7 dpi; 9, leaf epidermis of inoculation group in 12 dpi. **(B)** Cultural feature of gfp-tagged *K. aerogenes* HGG15 re-isolated from mulberry on LB medium containing 50 μg/mL kanamycin. **(C)** Observation of re-isolated gfp-tagged *K. aerogenes* HGG15 strain under green fluorescence at 488 nm. **(D)** Population dynamics of gfp-tagged *K. aerogenes* HGG15 in mulberry seedling.

To examine the colonization characteristics of *K. aerogenes* HGG15 in mulberry seedlings, plasmid pGFP4412 containing a *gfp* marker gene was transferred into wild type strain to generate *K. aerogenes* HGG15/gfp strain (Supplementary Figures 2A,B), which exhibited green fluorescence (Supplementary Figure 2C). This strain could be detected in various tissues of mulberry seedling by fluorescence microscopy after inoculation. *K. aerogenes* HGG15/gfp first formed bacterial aggregates at the sites of root hair emergence (Figure 4A1) and colonized on the plant lateral roots (Figure 4A2). It subsequently proceeded to enter the plant root epidermis (Figure 4A3) after about 1–2 day post infection (dpi) where many gfp-tagged bacterial cells could be observed in the cortex of stem tissue by 3–5 dpi (Figures 4A4–6). As time progressed, a few colonies could be seen in the petiole and leaf (Figures 4A7–9). As expected, no gfp-labeled cells were ever observed in mulberry seedlings treated with sterile water (Figures 4Ar,s,I).

The quantification of *K. aerogenes* HGG15/gfp within plant tissues was performed by selective culturing of plant macerates since the strain had the capability to grow on the LB plates containing 50 μg/mL kanamycin (Figure 4B) and exhibited green fluorescence (Figure 4C). The numbers of *K. aerogenes* HGG15/gfp decreased progressively from mulberry roots to the stems and leaves. The localization and distribution of *K. aerogenes* HGG15/gfp within the plants changed with time (Figure 4D). The amount of bacteria in roots gradually decreased after initially multiplying rapidly there. Population size of fresh root tissue was 8.37 and 5.42 lg CFU/g at 3 and 21 dpi, respectively. A similar temporal pattern of colonization of stems and leaves was also seen. Total of 7.65 lg CFU/g in stems and 7.21 lg CFU/g in leaves were initially seen and dropped to 4.15 lg CFU/g in stems and 3.94 lg CFU/g in leaves by 21 dpi, respectively.

### Mulberry-associated microbiome is influenced by *Klebsiella aerogenes* HGG15

**FIGURE 5:**
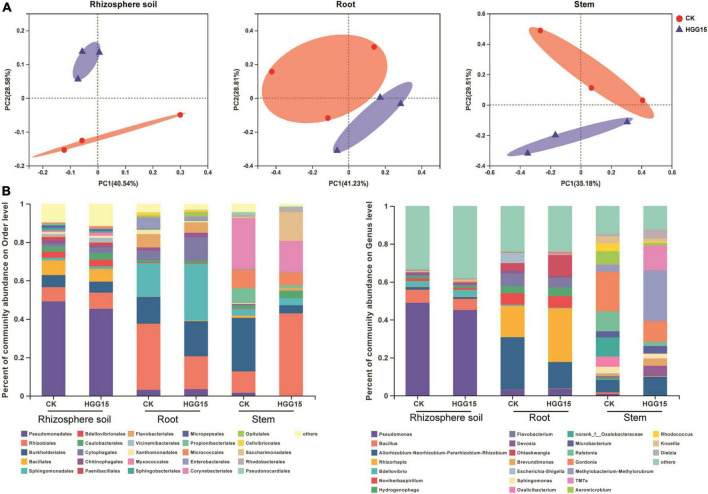
Effect of *K. aerogenes* HGG15 strain on mulberry associated bacterial communities. **(A)** Principal coordinate analysis of bacterial communities in the rhizosphere soil, root and stem of mulberry based on Bray–Curtis distances. **(B)** Composition of bacterial communities in the rhizosphere soil, root and stem of mulberry at order (left) and genus (right) level. Taxa with an abundance < 0.01 were included in “others.” Each column represented the mean of three biological replicates.

Bacterial community composition of mulberry treated with *K. aerogenes* HGG15 was compared to that of control plants by 16S ribosomal RNA amplicon analysis. Complete data sets were submitted to the NCBI SRA database (Accession Number: SRP367158). All rarefaction curves tended to reach a plateau (Supplementary Figure 3), indicating that the depth of sequencing was sufficient. Changes in α and β diversity were assessed to detect the effects of *K. aerogenes* HGG15 on mulberry-associated bacterial communities. Alpha diversity assessed by Sob and Shannon indexes was not significantly different between control and *K. aerogenes* HGG15-treated plants, whereas α diversity strongly varied between different tissues (Supplementary Figure 4A). As expected, the α diversity was highest in rhizosphere soils and lowest in stems. Moreover, the number of shared OTUs and unique OTUs showed a trend of gradual decline from rhizosphere soil to stem (Supplementary Figure 4B). In addition, the results of β diversity analysis as revealed by PCoA showed that bacterial communities mainly formed two separate clusters with the distance between inoculated samples being smaller than between controls (Figure 5A), although the statistical differences of groups were not significant according to PERMANOVA analyses (rhizosphere soil: *P* = 0.2, *R*^2^ = 0.3; root: *P* = 0.1, *R*^2^ = 0.4; stem: *P* = 0.2, *R*^2^ = 0.3). These results indicated that *K. aerogenes* HGG15 strain might impact the bacterial community regardless of whether it was the rhizosphere or endosphere.

To further understand the influence of *K. aerogenes* HGG15 on the shifts of microbial communities, the relative abundance of various bacterial taxa was analyzed. *K. aerogenes* HGG15 altered the composition of bacterial communities in root and stem, however, little differences between the control and inoculation groups were seen in the rhizosphere soil (Figure 5B). At the order level, the relative abundance of Sphingomonadales (HGG15: 29.53%; CK: 17.56%) and Cytophagales (HGG15: 12.15%; CK: 4.75%) were increased in inoculated roots, while the relative abundance of Rhizobiales (HGG15: 17.09%; CK: 34.50%) was decreased (Figure 5B). Moreover, Rhizobiales (HGG15: 42.88%; CK: 11.24%) and Saccharimonadales (HGG15: 15.02%; CK: 0.67%) were more abundant in stems of inoculated plants. In contrast, Pseudomonadales (HGG15: 0.13%; CK: 1.57%), Burkholderiales (HGG15: 4.24%; CK: 27.87%), Propionibacteriales (HGG15: 1.41%; CK: 7.42%), and Corynebacteriales (HGG15: 16.17%; CK: 26.29%) were enriched in stems of control plants. At the genus level, the roots of inoculated plants harbored significantly more *Ohtaekwangia* (HGG15: 11.09%; CK: 3.89%) and *Rhizorhapis* (HGG15: 28.25%; CK: 16.60%) than control plants (Figure 5B). *Rhizobium* (HGG15: 9.88%; CK: 6.52%), *Devosia* (HGG15: 5.52%; CK: 0.67%), *Brevundimonas* (HGG15: 3.72%; CK: 1.12%), *Methylorubrum* (HGG15: 26.70%; CK: 3.60%), and *TM7a* (HGG15: 13.22%; CK: 0.22%) were the predominant genera in the stems of *K. aerogenes* HGG15 inoculated plants, while the relative abundance of *Oxalicibacterium* (HGG15: 0%; CK: 5.39%), *norank_f_Oxalobacteraceae* (HGG15: 0%; CK: 10.11%), *Ralstonia* (HGG15: 2.31%; CK: 10.56%), *Aeromicrobium* (HGG15: 1.28%; CK: 6.97%), and *Rhodococcus* (HGG15: 0.51%; CK: 4.04%) were lower than in control plants (Figure 5B). These results revealed that *K. aerogenes* HGG15 substantially shifted endophytic bacterial composition of mulberry seedlings, while it had little effect on bacterial communities in rhizosphere soil.

**FIGURE 6:**
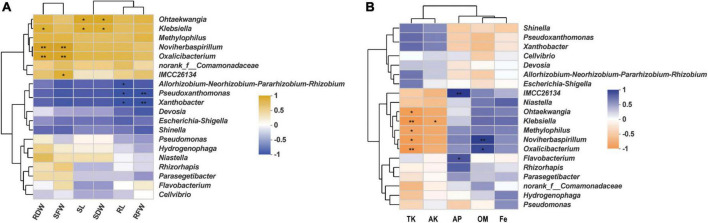
The relationship of root bacterial genera and mulberry development and soil properties. **(A)** Heatmap of correlation between mulberry growth parameters and top 20 bacterial genera using the Spearman correlation coefficient. RDW and SDW represented the dry weight of mulberry root and shoot, respectively. RFW and SFW represented the fresh weight of mulberry root and shoot, respectively. SL and RL represented mulberry shoot length and main root length, respectively. **(B)** Heatmap of correlation between soil properties and top 20 bacterial genera using the Spearman correlation coefficient. TK, AK, AP, OM, and Fe represented total potassium, available potassium, available phosphorus, organic matter, and available iron, respectively.

In order to uncover any significant differences in endophytic communities between *K. aerogenes* HGG15-treated and control plants, the Wilcoxon rank-sum test of bacterial community composition was investigated at the genus level. The relative frequency of *Xanthobacter* and *o_norank_c__Alphaproteobacteria* was significantly lower (*P* < 0.05) in roots of *K. aerogenes* HGG15 treated plants than in controls, while the proportion of *Methyloversatilis* and *MND1* was higher (*P* < 0.05) in treated plants (Supplementary Figure 5A). Notably, the genus of *Klebsiella* was relatively abundant in mulberry roots after inoculating *K. aerogenes* HGG15 (*P* < 0.01), while it was hardly detected in the rhizosphere soil and stem tissues (Supplementary Figure 5B), suggesting *Klebsiella* specifically colonized on mulberry roots. The Spearman correlation of the top 20 bacterial genera in root with mulberry growth indexes (Supplementary Table 4) was further investigated to uncover role of the *Klebsiella* genus. Results revealed that there was a positive relationship between *Klebsiella* and mulberry development, especially in root dry weight, shoot dry weight, and shoot length (*P* < 0.05) (Figure 6A), indicating that *Klebsiella* played a crucial role in promoting mulberry growth. In addition, soil property analysis showed that the content of organic matter was significantly higher in *K. aerogenes* HGG15 group (*P* < 0.01) (Supplementary Figure 6B), and potassium content was lower compared to that in the control group (Supplementary Figures 6C,D) (*P* < 0.01), but the contents of organic carbon, available phosphorus, and iron elements did not show dramatic variation (Supplementary Figures 6A,E,F). Furthermore, a heatmap constructed using the Spearman correlation coefficients was used to determine how the various microbial communities correlated with soil factors, revealing that *Klebsiella* was positively linked to total potassium and available potassium (Figure 6B). Altogether, these results suggested that *K. aerogenes* HGG15 strain significantly affected mulberry endophytic bacterial community, especially in root, and that the abundance of *Klebsiella* genus in root exhibited positive correlations with mulberry development and potassium content of soil, suggesting *Klebsiella* might contribute to mulberry seedling growth.

### *Klebsiella aerogenes* HGG15 affects the mulberry metabolite profile

**FIGURE 7:**
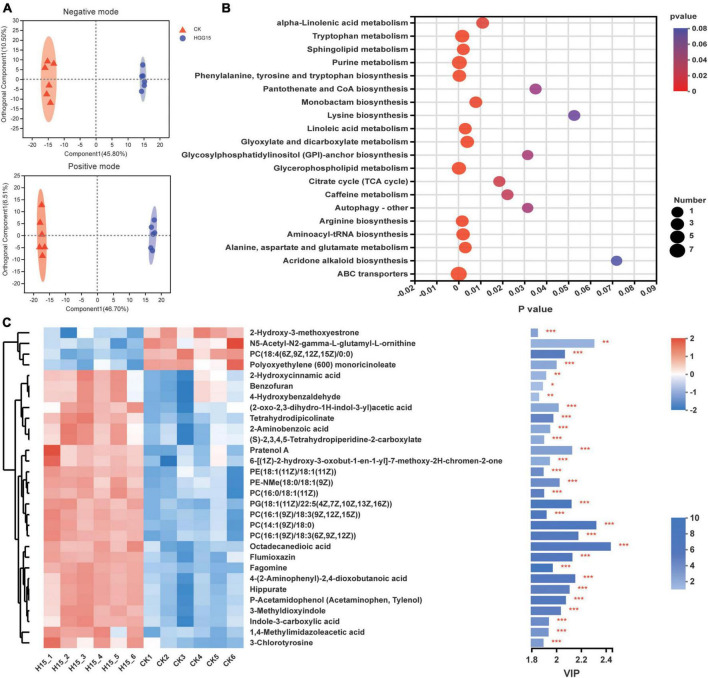
Effects of *K. aerogenes* HGG15 strain on metabolites of mulberry root. **(A)** Orthogonal partial least squares discriminant analysis of mulberry metabolites using positive and negative modes. **(B)** KEGG pathway enrichment analysis of differentially accumulated metabolites. The *x*-axis and *y*-axis represented the *P*-value of enrichment and enrichment pathway, respectively. The size of node represented the amount of metabolite enriched in the pathway. **(C)** Variable importance in projection scores of top 30 different metabolites between control and inoculated group. Left, heatmap of the differentially accumulated metabolites. The correlation of color and value was shown in the upper right bar. Right, bar chart of differentially accumulated metabolites. The length of bar indicated the contribution degree of this metabolite. The color of bar showed the difference degree of metabolite and the darker color represented the larger value of −log_10_(*P*-value) as shown in lower right bar. ***P < 0.001, **P < 0.01, *P < 0.05.

Since *K. aerogenes* HGG15 colonized on the interior of mulberry roots (Figure 4D) and promoted plant growth, and the genus *Klebsiella* is also a dominant colonizer in mulberry roots (Supplementary Figure 5B), untargeted metabolomics of roots were analyzed to determine if and how this strain affected mulberry metabolites. PCA of metabolites demonstrated that inoculated and control plants differed in their metabolome regardless of negative mode or positive mode of metabolite analysis (Supplementary Figure 7A). The metabolite profiles of mulberry were then subjected to OPLS-DA, which showed difference between the control and *K. aerogenes* HGG15-treated group in both negative and positive analytic modes (Figure 7A). The score plots of PCA and OPLS-DA exhibited an obvious separation between the control and treatment groups, indicating *K. aerogenes* HGG15 was responsible for the distinction in categories and quantities of metabolites in mulberry. In addition, little variation among the biological replicates of each group was observed (Figure 7A and Supplementary Figure 7A), which illustrated the sufficient reproducibility and reliability of the experiment.

Metabolites with a threshold *P*-value < 0.05 and VIP > 1 were considered as differentially accumulated metabolites (DAMs). In total, there were 201 different identifiable metabolites, consisting of 125 and 76 metabolites from the positive and negative ionization modes, where 131 and 70 metabolites were upregulated and downregulated, respectively (Supplementary Figure 7B). According to their molecular structure and function based on the HMDB, the DAMs were classified into eight categories at the superclass level. The most dominant group of DAMs was lipids and lipid-like molecules which contained glycerophospholipids, fatty acyls, glycerolipids, sphingolipids, and prenol lipids. The other DAMs were classified into organoheterocyclic compounds, organic acids and derivatives, organic oxygen compounds, phenylpropanoids and polyketides, nucleosides, nucleotides, and analogues, benzenoids, and organosulfur compounds (Table 2). At the class level, the content of 25 category substances (78.13%, occupying 32 categories), such as glycerolipid, sphingolipid, indole, and pyridine, were significantly upregulated in mulberry after *K. aerogenes* HGG15 treatment (Table 2).

**TABLE 2:** The classification of significantly different metabolites of mulberry root.

Superclass	Class	Regulate	Number
Lipids and lipid-like molecules	Glycerophospholipids	Up/down	52
	Fatty acyls	Up/down	21
	Steroids and steroid derivatives	Up/down	4
	Glycerolipids	Up	4
	Sphingolipids	Up	2
	Prenol lipids	Down	1
Organoheterocyclic compounds	Pyridines and derivatives	Up	4
	Indoles and derivatives	Up	3
	Piperidines	Up	3
	Tetrahydrofurans	Up/down	2
	Benzofurans	Up	2
	Imidazopyrimidines	Up	2
	Azacyclic compounds	Up	1
	Furans	Up	1
	Diazines	Up	1
	Benzoxazines	Up	1
	Benzothiophenes	Down	1
	Benzopyrans	Up	1
Organic acids and derivatives	Carboxylic acids and derivatives	Up/down	19
	Keto acids and derivatives	Down	1
Organic oxygen compounds	Organooxygen compounds	Up/down	11
Phenylpropanoids and polyketides	Cinnamic acids and derivatives	Up	2
	Flavonoids	Down	2
	Stilbenes	Down	1
	Isocoumarins and derivatives	Up	1
	Coumarins and derivatives	Up	1
Nucleosides, nucleotides, and analogues	Purine nucleotides	Up/down	2
	Purine nucleosides	Up	2
Benzenoids	Phenols	Up	2
	Benzene and substituted derivatives	Up	1
Organosulfur compounds	Thioethers	Down	1

To further understand the DAMs function and the related biological processes they participated in, the top 20 pathways enrichment analysis of the DAMs was conducted using KEGG. The results showed that the ABC transporter, glycerophospholipid metabolism, purine metabolism, arginine biosynthesis, and tryptophan metabolism were significantly enriched (Figure 7B). Most of these pathways are involved in basic biological functions of plants, indicating that primary metabolism in mulberry was significantly affected by *K. aerogenes* HGG15. Furthermore, the VIP values of DEMs were calculated to explore the specifically different metabolites in mulberry. As shown in Figure 7C, the metabolites 2-hydroxy-3-methoxyestrone, PC [18:4(6Z,9Z,12Z,15Z)/0:0], N5-acetyl-N2-gamma-L-glutamyl-L-ornithine, and polyoxyethylene (600) monoricinoleate were significantly decreased in roots after *K. aerogenes* HGG15 treatment. In contrast, some compounds, e.g., octadecanedioic acid, 4-(2-aminophenyl)-2,4-dioxobutanoic acid, pratenol A, flumioxazin, hippurate, 3-methyldioxyindole, tetrahydrodipicolinate, fagomine, 2-aminobenzoic acid, and indole-3-carboxylic acid exhibited strong positive correlations with *K. aerogenes* HGG15 treatment. These results illustrated that the metabolites of mulberry root were strongly influenced by *K. aerogenes* HGG15, likely explaining its large effect on mulberry growth. In addition, a heatmap of correlation was generated to explore the potential relationship between differential bacteria altered by *K. aerogenes* HGG15 and metabolites of mulberry root. As expected, the metabolites were closely associated with differential bacterial microbiome of mulberry roots (Supplementary Figure 8). In particular, a greatly significant correlation has appeared between DAMs and bacterial taxa such as *o_norank_c_Alphaproteobacteria*, *Klebsiella*, *Methyloversatilis*, *Reyranella*, *Ammoniphilus*, *Conexibacter*, and *Ohtaekwangia*. Thus, differential bacteria altered by *K. aerogenes* HGG15 could further influence mulberry metabolites, where the synergistic linkage of microbiome and metabolites might together contribute to the development of host plant.

## Discussion

Abiotic stresses are the foremost limiting factors for agricultural productivity. Various strategies including transgenic technology and molecular breeding have been considered to assist plant to relieve the stresses of such adverse conditions created by environmental extremes. Such approaches are often time consuming and might meet societal pressures. The modulation of endophytic colonization of plants is gaining wide popularity as an alternate strategy for improving stress tolerance of plants (Glick, 2014; Tiwari et al., 2016), since endophytes could provide sustainable benefits of both improving plant tolerance to biotic or abiotic stresses as well as stimulating plant growth (Bacon and White, 2015; Zhang et al., 2019). Therefore, the application of endophytes could be a potential method for enhancement of mulberry growth in the hydro-fluctuation belt of the TGR. Bacteria in the genera of *Enterobacter*, *Pantoea*, and *Klebsiella* have been the most extensively studied taxa and have been shown to benefit plant development (Ullah et al., 2019; Xu S. D. et al., 2022). Earlier studies reported that *Enterobacter* sp. E5 increased resistance of banana to *Fusarium* wilt disease (Liu et al., 2019). Zhang et al. (2017) also revealed that the endophyte *Klebsiella* sp. LTGPAF-6F could contribute to both growth promotion and improvement of drought tolerance of wheat in greenhouse studies. In the present study, a total of 28 isolates from mulberry were screened as PGPBs and *Enterobacter* spp., *Pantoea* spp., and *Klebsiella* spp. were the dominant genera with traits, which might play crucial roles in endowing mulberry beneficial traits in hydro-fluctuation belt (Table 1). Among them, four isolates were selected for assessment their PGP traits *in planta* and showed excellent growth stimulating effects to mulberry seedlings regardless of whether they experienced flood stress conditions or not (Figures 1, 2), and strain *Klebsiella aerogenes* HGG15 was found to be much superior to these other strains.

The plant growth promoting effect of bacteria is correlated with its colonization of host plants. It makes sense that successful colonization is critical for impacts of endophytes and their host plants as has been seen in many studies (Jha and Kumar, 2007; Pavlova et al., 2017; Ullah et al., 2019). In this study, *K. aerogenes* HGG15/gfp initially attached to emerging root hairs and lateral roots, indicating that it likely invades plants through these natural sites. Hallmann and Berg (2006) showed that, like *K. aerogenes* HGG15/gfp, endophytic bacterial population were lower within above-ground tissues, e.g., stem and leaves than in roots. Interestingly, we also found through amplicon sequencing analysis that other *Klebsiella* also preferentially colonized on mulberry roots (Supplementary Figure 5B), which might be the reason why *K. aerogenes* HGG15 greatly influenced root biomass and architecture of mulberry (Figures 1, 2). It has been reported that some *Klebsiella*, such as *K. pneumonia*, are opportunistic pathogens of animals (Li and Huang, 2017; Álvarez-Marín et al., 2021), while major species of *Klebsiella* usually did not cause disease in plants. More importantly, most of *Klebsiella* such as *Klebsiella* sp. D5A (Liu et al., 2016) and *Klebsiella* sp. SBP-8 (Singh et al., 2015) could improve plant utilization of nitrogen, phosphorus, and iron, and contribute to biological control or environmental tolerance. This taxon thus has attracted attentions as a plant growth stimulator in agricultural systems. Notably, the results of our safety evaluation of *K. aerogenes* HGG15 revealed that it had no side effects on the silkworm. These results suggested that the endophytic bacterium *K. aerogenes* HGG15 might be a potential growth promoter for crop plants, but its biosafety will need further evaluation by determining haemolysis, cytotoxicity, antibiotic resistance and genotoxicity.

Inoculation of exogenous bacteria not only influences the microbiome structure of different plant niches (Li et al., 2021), but also can attract certain beneficial bacteria, such as *Rhizobium* (Guo and Chi, 2014), *Methylorubrum* (Alessa et al., 2021), and *Microbacterium* (Vilchez et al., 2018), to promote plant growth and inhibit disease occurrence (Zhuang et al., 2021). We explored the bacterial community in different compartments of mulberry and found that bacterial β diversity in rhizosphere soil, root, and stem of mulberry was influenced by *K. aerogenes* HGG15 (Figure 5A), while there was no obvious effect on α diversity (Supplementary Figure 4A). The taxonomic composition of endophytic bacteria exhibited differences between the control and inoculated plants, especially those inhabiting in root and stem, but there was little effect of inoculation on rhizosphere soil communities (Figure 5B). *Methylorubrum* was the preponderant bacterial endophyte appearing in *K. aerogenes-*inoculated mulberry. Similar findings were found in soybean (Hara et al., 2019) and pine seeds (Koskimäki et al., 2022), which were dominantly colonized by *Methylorubrum* and exhibited higher biomass of shoots and roots. This taxon may thus contribute to mulberry growth. Notably, *Ralstonia*, one of the most destructive plant bacterial pathogens (Pan et al., 2013), was markedly decreased in mulberry after inoculation. In addition, compared with control plants, roots of mulberry treated with *K. aerogenes* HGG15 accumulated significantly less *Xanthobacter* (Supplementary Figure 5A), a potential phytopathogen causing serious disease in cruciferous plants (Mansfield et al., 2012). These results indicated that *K. aerogenes* HGG15 may play a crucial role in antagonizing against pathogens as well as is a good candidate strain as bio-fertilizer, but its antagonistic efficiency should be further studied.

In the process of bacteria-assisted plant growth promotion, some material exchange, energy transformation, and information communication continuously take place among microbes, plant roots, and the soil environments (Sun L. J. et al., 2020; Sun et al., 2022). In our study, the increased content of organic matter and decreased content of potassium in soils of mulberry inoculated with *K. aerogenes* HGG15 were found (Supplementary Figure 6), indicating a feedback effect of this strain on soil processes. This finding is in accordance with previous study that physicochemical properties of soils of *Brassica juncea* were strongly influenced by endophytic bacterial *Serratia marcescens* PRE01 and *Arthrobacter ginsengisoli* PRE05 (Wang et al., 2020). Interestingly, we found that *K. aerogenes* HGG15 strain had a capability to dissolve unavailable potassium *in vitro* (Supplementary Figure 9), suggesting that this strain could transform the unavailable potassium to available form, and thus assisted mulberry trees uptake useful potassium from soil to benefit their growth. Metabolites in plant roots usually exert a variety of functions, such as facilitating primary metabolism and root growth, rhizosphere communication, and plant defense (Doell et al., 2021). Mutualistic interactions of plant with endophyte can facilitate plant growth promotion through the accumulation of beneficial plant metabolites (Kundu et al., 2021), as in the case of fungus *Piriformospora indica* that colonize in Chinese cabbage, which stimulated plant synthesis of metabolites involved in the tryptophan and phenylalanine metabolism as well as γ-aminobutyrate (Hua et al., 2017). Furthermore, *Bacillus amyloliquefaciens* enhanced maize growth by improving nutrient uptake and influencing plant primary metabolism, especially glucose, fructose, alanine and γ-aminobutyric acid metabolism (Vinci et al., 2018). Most differentially abundant compounds found in our study were annotated as lipids and lipid-like molecules (Table 2), especially glycerolipids and sphingolipids, which not only contributed to abiotic and biotic stress resistance but also regulated basically cellular processes (Luttgeharm et al., 2016). It was worth noting that we found the production of indole derivatives affiliated with organoheterocyclic compounds in mulberry was elevated by *K. aerogenes.* These compounds have been shown to enhance lateral root growth (Sun X. et al., 2020). Additionally, the indole-3-carboxylic acid, one of the indole derivatives, is the best known of the auxins, which is involved in camalexin biosynthesis (Wang et al., 2012) and plays an important role in plant pathogen defense (Magnus et al., 1980). *K. aerogenes* HGG15 enhanced the content of indole-3-carboxylic acid in mulberry roots, suggesting that this strain might induce resistance of host plant to biotic or abiotic stress. Notably, cinnamic acids, isocoumarins, coumarins, and their derivatives, as important phenylpropanoid and polyketide compounds, were significantly upregulated in mulberry after *K. aerogenes* HGG15 inoculation (Table 2). Among them, the downstream substance of cinnamic acid has been described as a potential antioxidant compound in mulberry (Park et al., 2017), which might be beneficial for mulberry development under abiotic stress conditions. Coumarins are known to play a key role in the transport of iron-mobilization in plant and are recognized as plant signals shaping host microbiomes (Stringlis et al., 2018). Moreover, coumarins and their derivatives have been extensively studied for their potential to help plants cope with biotic and abiotic environmental stress (Stringlis et al., 2019). Therefore, the above-mentioned metabolites might be crucial drivers of mulberry growth promotion and its enhanced stress resistance when inoculated with *K. aerogenes* HGG15.

## Conclusion

The growth of mulberry trees is greatly hindered by flood stress and limited-nutrition in the hydro-fluctuation belt of the TGR region. In the current study, *Klebsiella aerogenes* HGG15 was ultimately screened out as having superior plant growth promotion proprieties *in vitro* and strongly stimulated growth and enhanced flood tolerance of mulberry *in planta*. Moreover, it was not harmful to silkworm and could extensively and persistently colonize on mulberry. The inoculation of *K. aerogenes* HGG15 greatly altered the establishment of endophytic bacterial communities in mulberry, especially in roots and stems. In addition, the large change in abundance of abiotic stress response factors and compounds that promote plant growth such as glycerolipid, sphingolipid, indole, pyridine, and coumarin were observed in inoculated mulberry. Taken together, these results of this study help us to understand the interactions between this endophyte and plants and mechanisms driving these interactions, and provide innovative approaches for revegetation in the hydro-fluctuation belt through enhancing microbe-assisted plant growth.

## Data availability statement

The datasets presented in this study can be found in online repositories. The names of the repository/repositories and accession number(s) can be found below: https://www.ncbi.nlm.nih.gov/genbank/, SRP367158; ON786677–ON786703; ON090422.

## Author contributions

TO and JX designed the experiments. TO, HG, KJ, JY, and RZ performed the experiments. TO analyzed and wrote the manuscript. JX and XL revised the manuscript. ZZ and ZX conceived the study and contributed resources. All authors contributed to the article and approved the submitted version.

## Footnotes

1
http://www.arb-silva.de

2
http://www.hmdb.ca/

3
https://metlin.scripps.edu/

4
https://docs.scipy.org/doc/scipy/
