# Supplementary material for: Endophytic Klebsiella aerogenes HGG15 stimulates mulberry growth in hydro-fluctuation belt and the potential mechanisms as revealed by microbiome and metabolomics
Source: Front Microbiol. 2022 Aug 12;13:978550. doi: 10.3389/fmicb.2022.978550 (PMC9417544; doi:10.3389/fmicb.2022.978550)
Supplement: Supplementary file 1 [file Data_Sheet_1.docx]

**TABLE S1│**Characteristics of mulberry trees in the hydro-fluctuation belt in Longjiao Town, Yunyang County, Chongqing Municipality, China.

|  | Wild mulberry | Newly planted mulberry |
| --- | --- | --- |
| Height (m) | 2.85 ± 0.34 | 3.79 ± 0.19 |
| Primary root diameter (cm) | 2.10 ± 0.10 | 2.95 ± 0.06 |
| Trunk diameter (cm) | 4.18 ± 0.83 | 6.50 ± 0.15 |
| Chlorophyll content (spad) | 24.03 ± 3.33 | 22.62 ± 2.99 |
| N content (mg/g) | 10.75 ± 0.31 | 10.30 ± 0.88 |

Values are the results of the mean ± standard deviations

**TABLE S2│**Number of endophytic bacterial isolates from well-growing mulberry trees in hydro-fluctuation belt.

| Sampling type | Tissue | Medium | | | | Total |
| --- | --- | --- | --- | --- | --- | --- |
|  |  | LB | TSA | NA | GA |  |
| Wild mulberry | Stem | 34 | 29 | 38 | 18 | 119 |
|  | Root | 25 | 31 | 24 | 8 | 88 |
| Newly planted mulberry | Stem | 19 | 15 | 23 | 11 | 68 |
|  | Root | 19 | 17 | 22 | 10 | 68 |

**TABLE S3│**Physiological and biochemical characteristics of the HGG15 strain.

| Test items | Results | Test items | Results |
| --- | --- | --- | --- |
| Mannitol | + | Arabinose | + |
| Voges-Proskauer | + | Urea | – |
| Glucose | + | Fructose | + |
| Lactose | – | H_2_S formation | + |
| Nitrate | + | Acetamide | + |
| Maltose | + | Semi-solid agar | + |
| Peptone water | – | Sucrose | + |

‘+’ represents positive (growth or reaction) and ‘–’ represents negative (no growth or no reaction).

**TABLE S4│**The mulberry growth parameters of Spearman correlation analysis.

| Treatment | RDW (mg) | RFW (mg) | RL (cm) | SDW (mg) | SFW (mg) | SL (cm) |
| --- | --- | --- | --- | --- | --- | --- |
| CK | 1.77±0.88 b | 12.25±4.99 b | 6.64±1.34 b | 29.15±18.15 b | 127.98±45.89 b | 12.22±0.52 b |
| HGG15 | 8.28±1.19 a | 36.25±6.17 a | 10.86±1.75 a | 82.84±20.00 a | 278.48±50.88 a | 16.65±2.54 a |

RDW and SDW represent the dry weight of mulberry root and shoot, respectively. RFW and SFW represent the fresh weight of mulberry root and shoot, respectively. SL and RL represent mulberry shoot length and main root length, respectively.


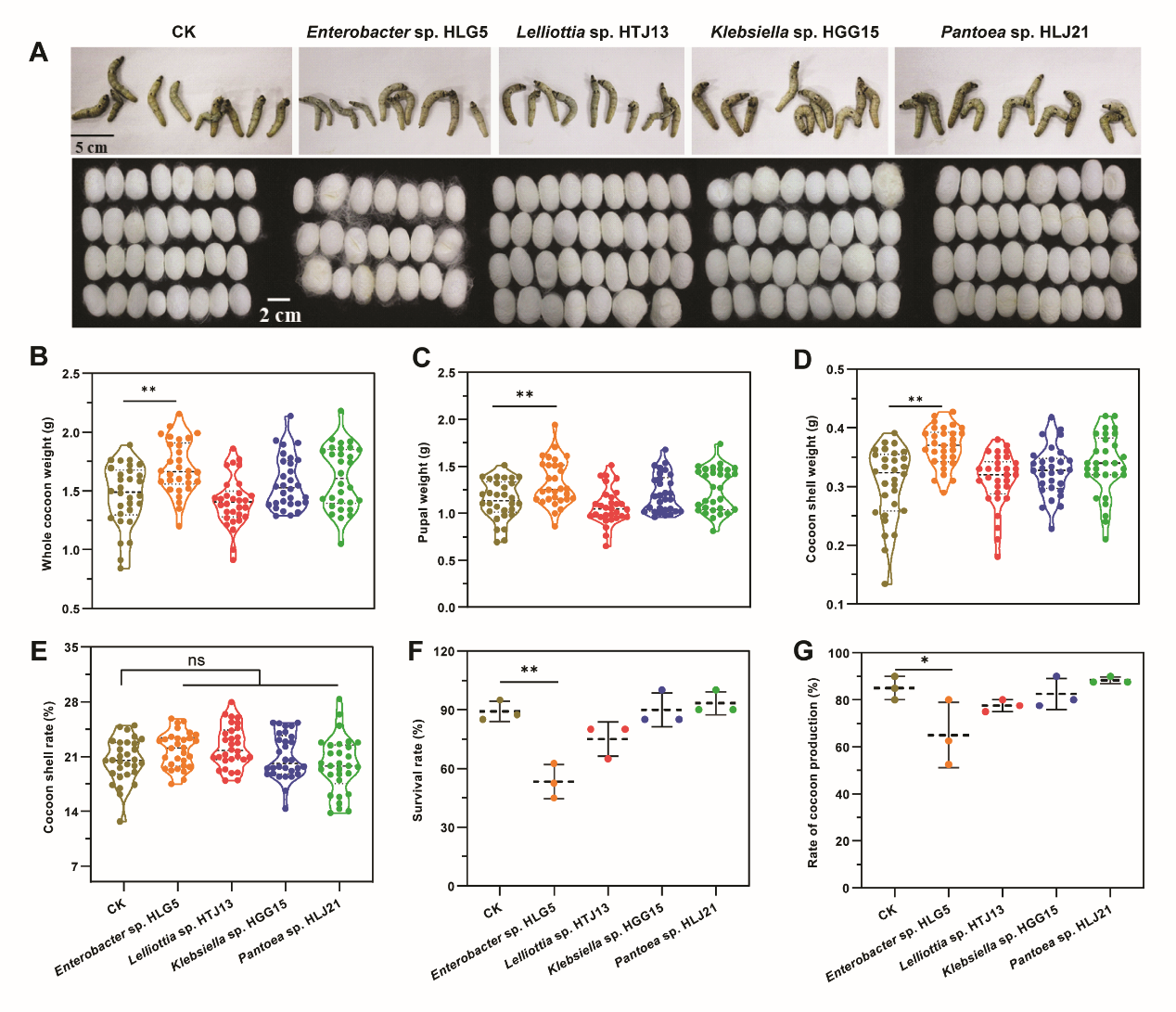


**FIGURE S1│**Bio-safety tests of potential four PGPBs on silkworm. (**A**) Representative photograph of silkworm at 5^th^ instar and cocoon. (**B**) Whole cocoon weight. (**C**) Pupal weight. (**D**) Cocoon shell weight. (**E**) Cocoon shell rate. (**F**) Survival rate of silkworm. (**G**) Rate of cocoon production. Data represented mean ± standard deviation (n = 30) (**A-E**). Data represented mean ± standard deviation of three biological replicates with 30 silkworms for each biological repeat (**F** and **G**). Significance between control and treatment groups was conducted by T-test. ** *P* < 0.01, * *P* < 0.05.


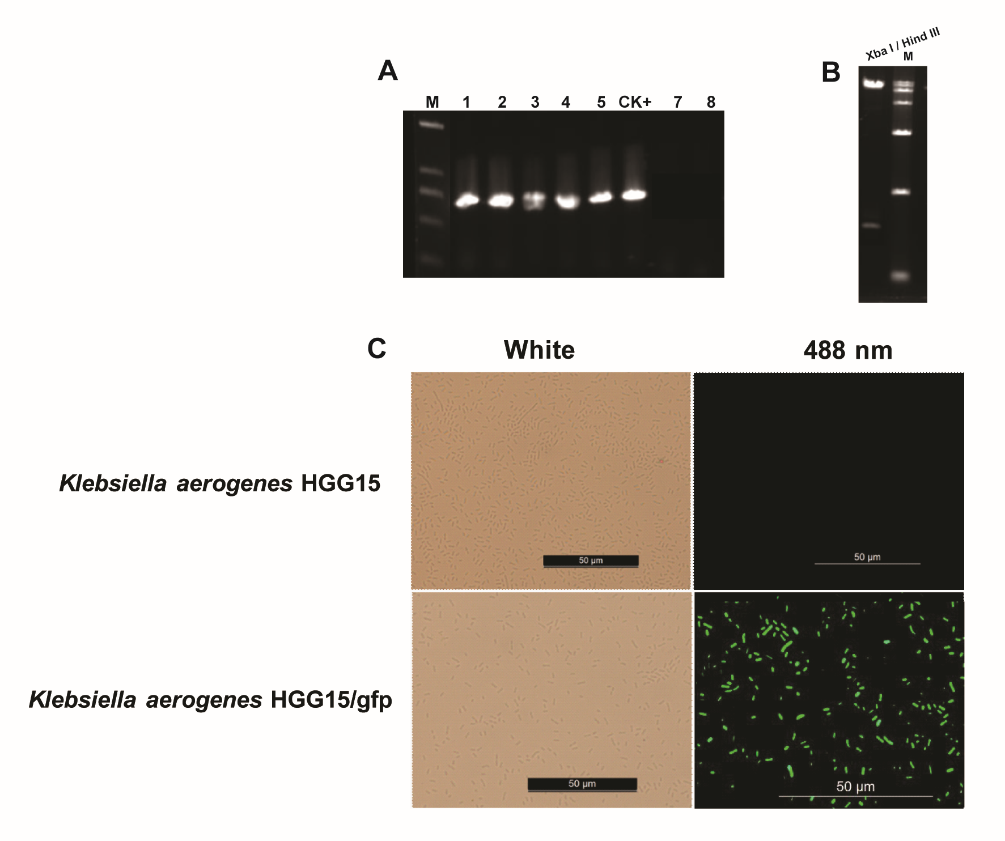


**FIGURE S2│**The construction of *K. aerogenes* HGG15/gfp strain. (**A**) Agarose gel electrophoresis of the PCR products amplified *gfp* gene from genomic DNA of *K. aerogenes* HGG15/gfp strains. Lane M, 2000 bp DNA marker. Lane 1-5, *K. aerogenes* HGG15 strain containing pGFP4412 plasmid. Lane CK+, pGFP4412 plasmid. Lane 7-8, wild type of *K. aerogenes* HGG15 strain. (**B**) Agarose gel electrophoresis of the plasmids extracted from *K. aerogenes* HGG15/gfp strain which were digested by XbaI and HindIII at 30℃ for 3 h. (**C**) Observation of gfp-tagged *K. aerogenes* HGG15 and wild type strains under white light and green fluorescence with 488 nm. The bacteria were cultured on LB medium at 30℃ for 24 h.


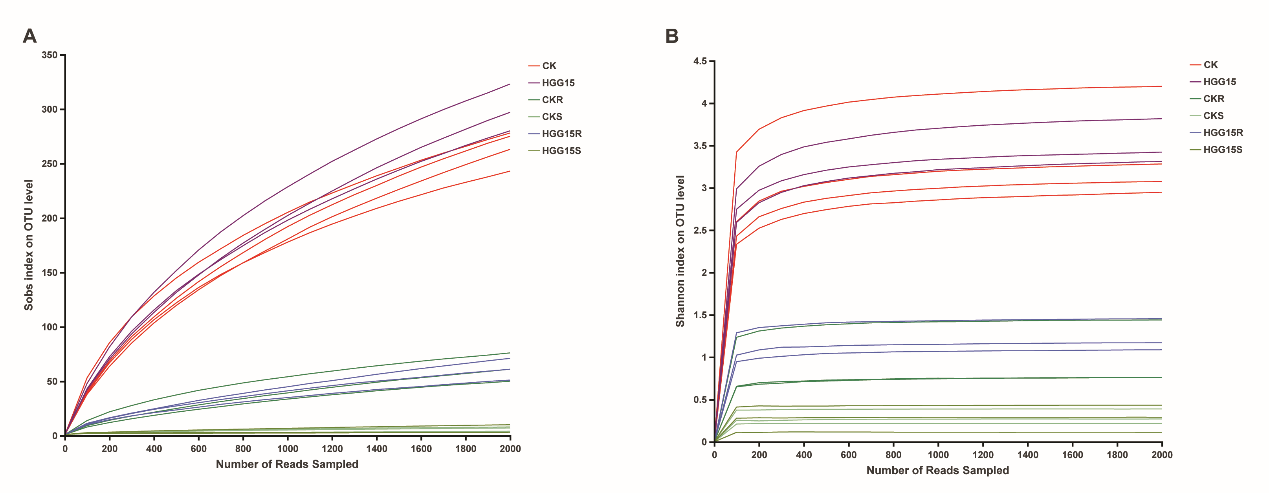


**FIGURE S3│** Rarefaction curves depicting the number of OTUs. (**A**) Rarefaction curve based on Sob index. (**B**) Rarefaction curve based on Shannon index. CK and HGG15 represented bacterial communities of rhizosphere soil from control group and *K. aerogenes* HGG15 inoculated group, respectively. CKR and HGG15R represented bacterial communities of root from control group and *K. aerogenes* HGG15 inoculated group, respectively. CKS and HGG15S represented bacterial communities of stem from control group and *K. aerogenes* HGG15 inoculated group, respectively.


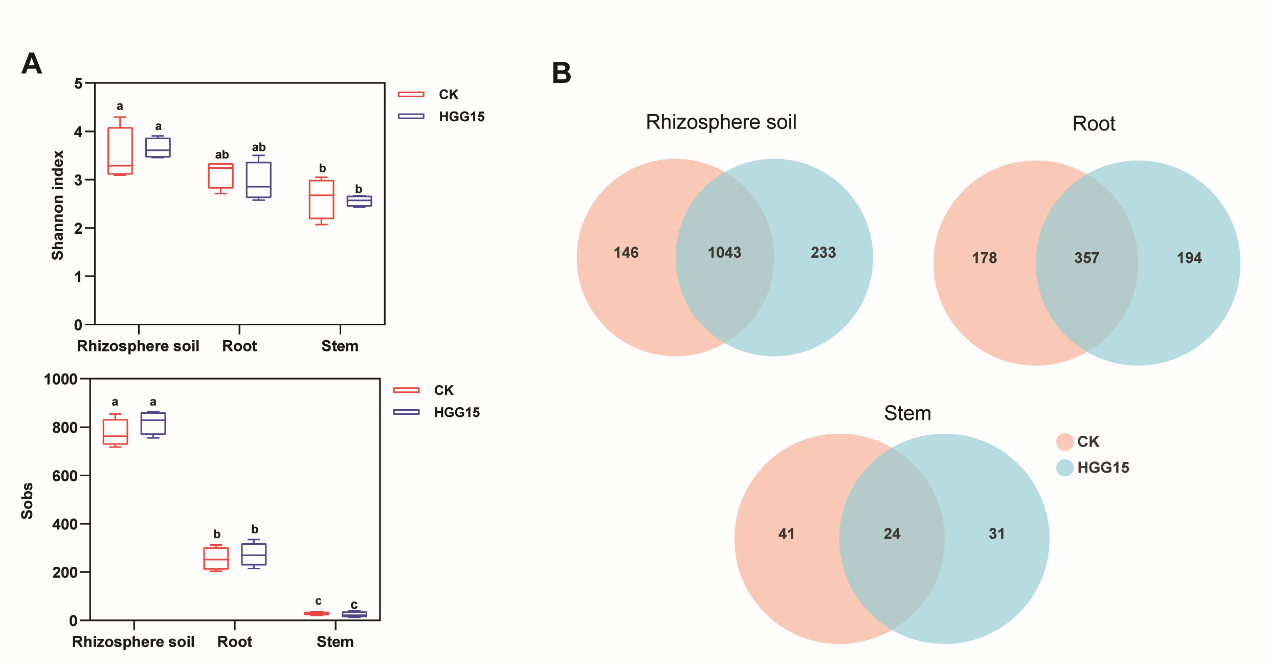


**FIGURE S4│**Effects of *K. aerogenes* HGG15 on mulberry associated bacterial communities. (**A**) α-diversity of mulberry different compartments between control and inoculated group. Data represented mean ± standard deviation (n = 3). Different letters indicated statistical differences using Tukey’s one-way ANOVA (*P* < 0.05). (**B**) Venn diagram of the number of OTUs obtained in different compartments between control and inoculated group. Values represented the number of OTUs.


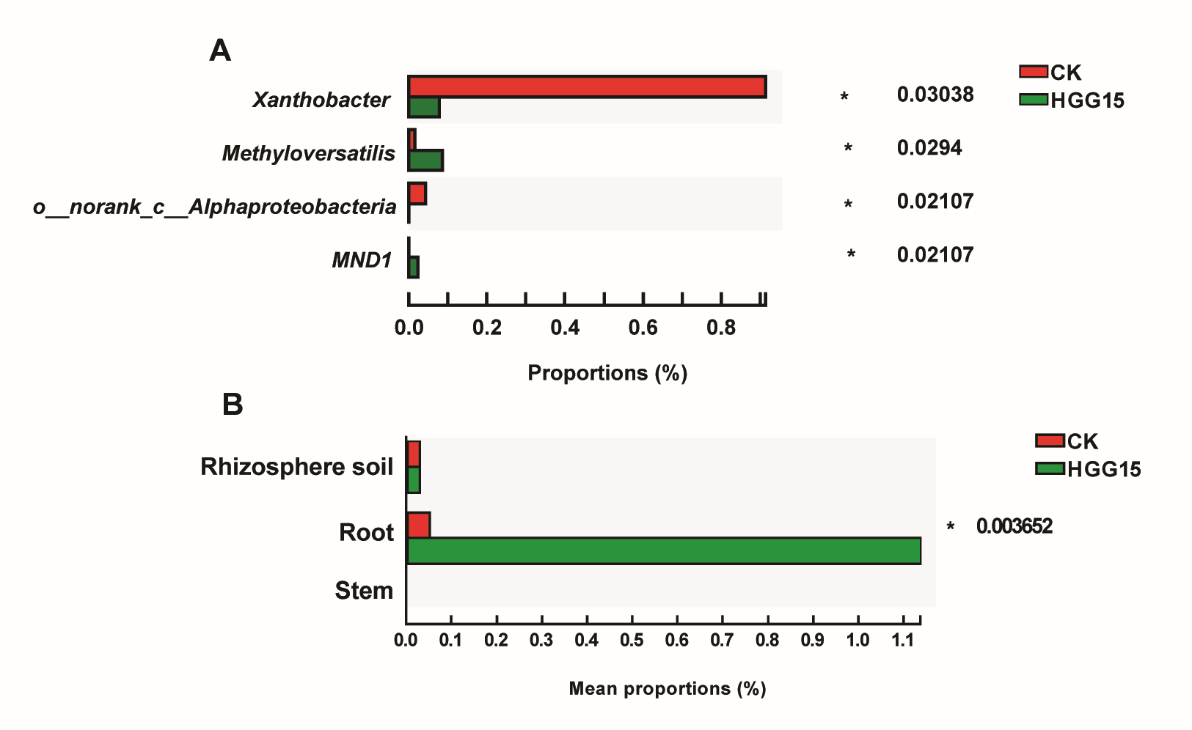


**FIGURE S5│**Comparisons in the relative abundance of the bacterial genera between control and *K. aerogenes* HGG15 group. (**A**) The significantly different genera in mulberry root. Statistical differences were conducted by Wilcoxon rank-sum test. Star letters indicated statistical differences. * *P* < 0.05. (**B**) The distribution of *Klebsiella* in mulberry different compartments. Star letters indicated statistical differences in the rhizosphere, root, and stem using Kruskal-Wallis H test. * *P* < 0.05.


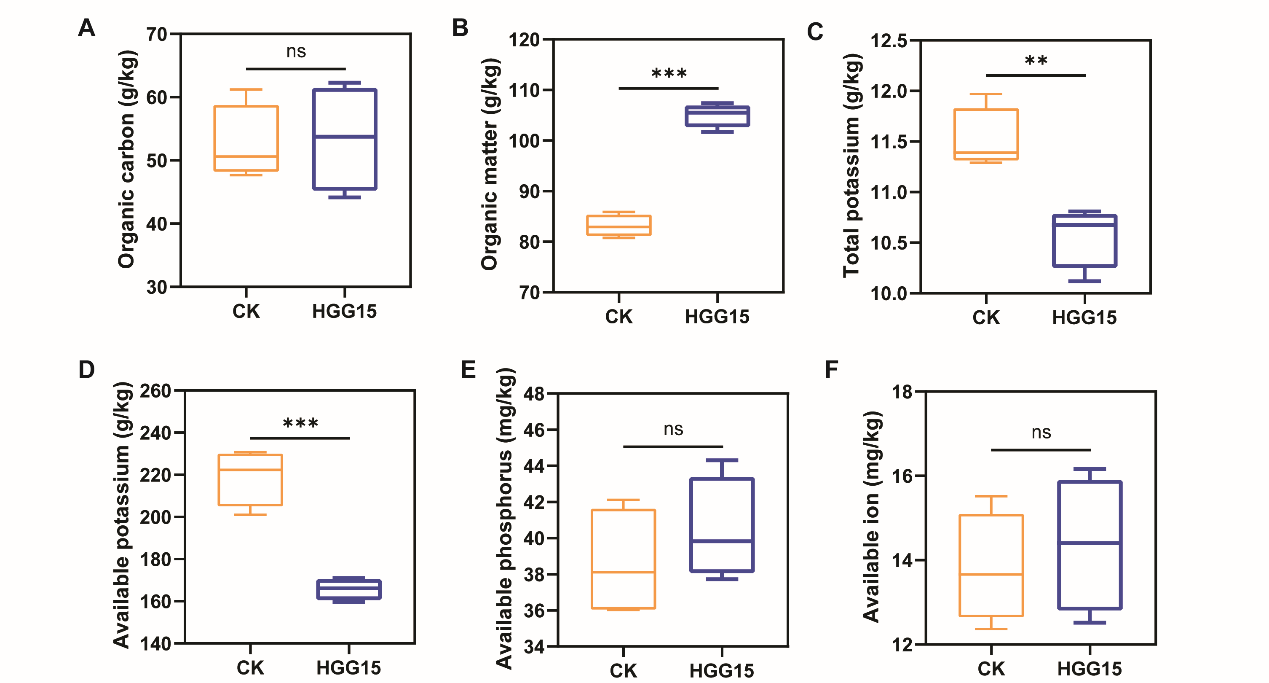


**FIGURE S6│**Effects of *K. aerogenes* HGG15 on the soil properties. Data represented mean ± standard deviation (n = 3). (**A**) Organic carbon; (**B**) Organic matter; (**C**) Total potassium; (**D**) Available potassium; (**E**) Available phosphorus; (**F**) Available ion. Statistical significance between control and treatment group was conducted by T-test. *** *P* < 0.001, ** *P* < 0.01.


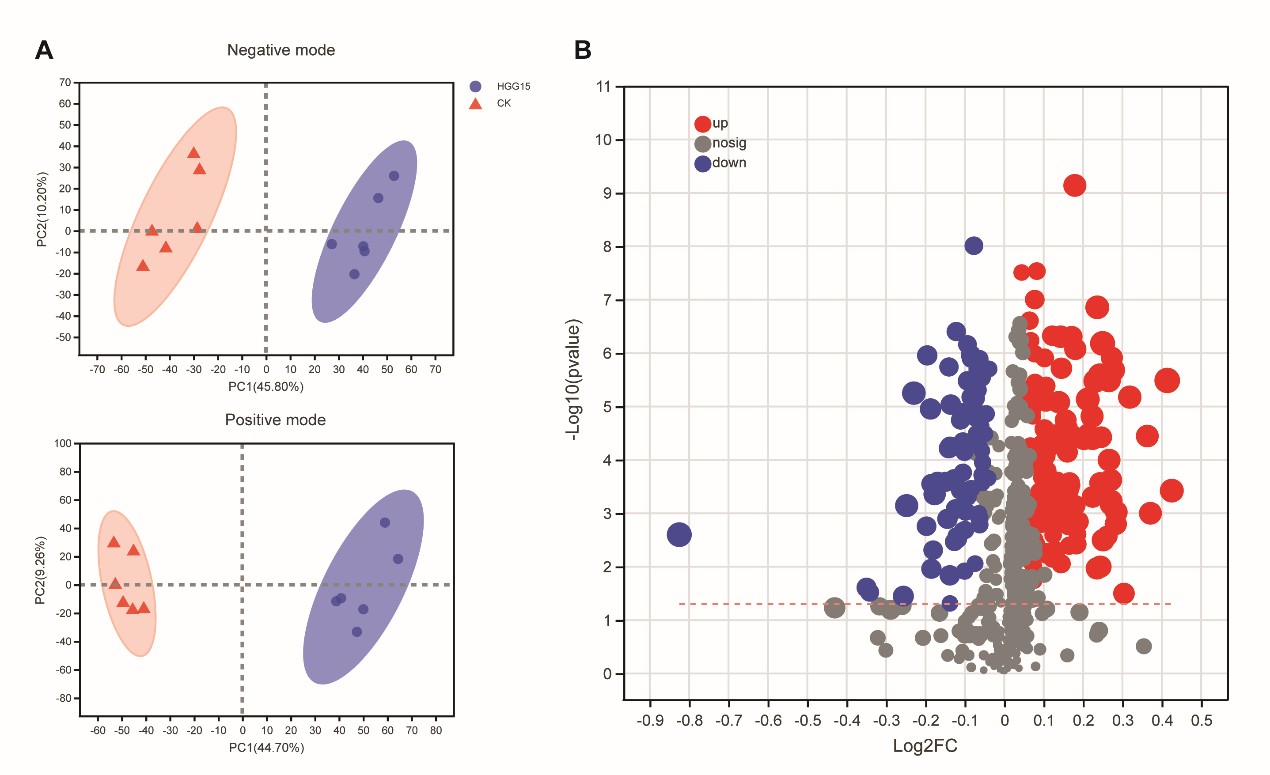


**FIGURE S7│**Comparisons of differentially accumulated metabolites (DAMs) between control and inoculated group. (**A**) Principal component analysis of DAMs using positive and negative modes using Student’s T-test analysis. (**B**) Volcano plot of DAMs in mulberry root. Blue, red, and gray dots represented downregulated, upregulated, and not altered DAMs, respectively. The size of node represented the VIP value of metabolite.


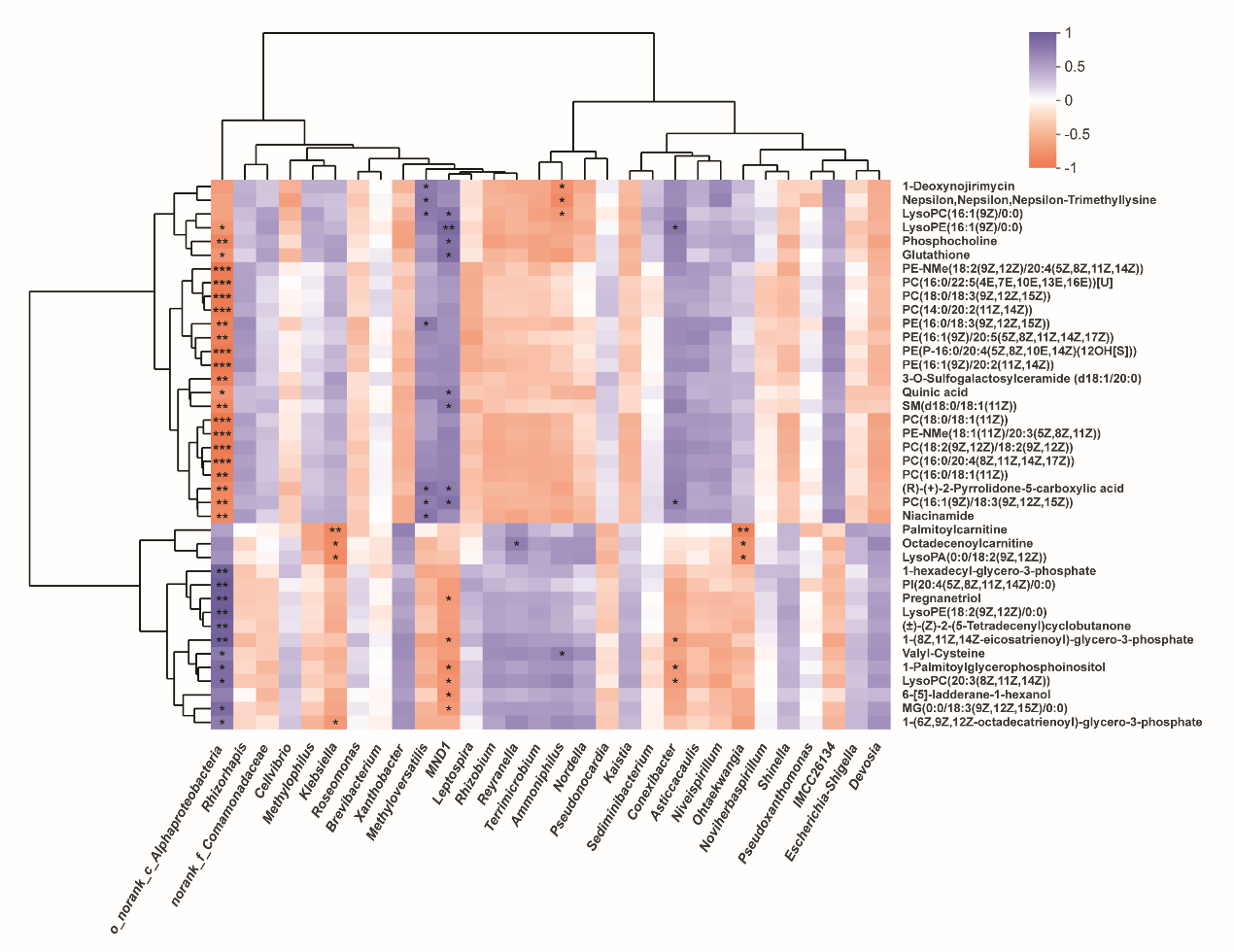


**FIGURE S8│**The heatmap of correlation between top 40 metabolites of mulberry root and bacterial genera using the Pearson correlation coefficient. *** *P* < 0.001, ** *P* < 0.01, * *P* < 0.05.


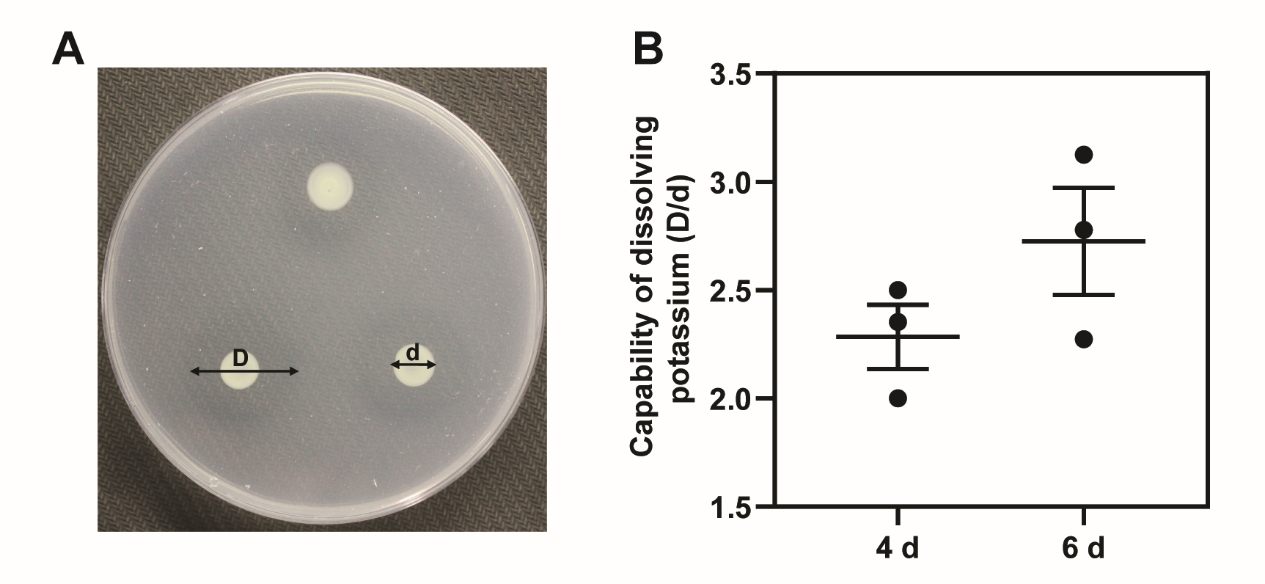


**FIGURE S9│**The dissolving potassium capability of *K. aerogenes* HGG15 strain. (**A**) *K. aerogenes* HGG15 inoculated in Alexandrov medium contained 5 g glucose, 2 g Na_2_HPO_4_, 0.5 g MgSO_4_.7H_2_O, 0.005 g FeCl_3_, 0.1 g CaCO_3_, and 1 g potassium feldspar powder and incubated at 37℃ for 4 days. D and d represented the diameter of hydrolysis circle and colony, respectively. (**B**) The potassium-dissolving ability after incubation 4 days and 6 days. Values represented the mean ± standard deviation of replicates (n = 3).
